# Effectiveness of Juniper Essential Oils in Reducing Selected Cigarette Smoke Toxicants, Improving Oxidative Parameters, and Cytotoxicity Against Lung Adenocarcinoma

**DOI:** 10.1155/bri/6238789

**Published:** 2025-08-17

**Authors:** Haouaouchi Fatma Zohra, Boudiba Sameh, Boudiba Louiza, Baya Berka, Karima Hanini, Gasmi Salim, Soraya Hioun, Alfred Ngenge Tamfu

**Affiliations:** ^1^Laboratory of Organic Materials and Heterochemistry (LOMH), Echahid Cheikh Larbi Tebessi University, Constantine Road, Tebessa 12002, Algeria; ^2^Laboratory of Applied Chemistry and Renewable Energies (LACRE), Echahid Cheikh Larbi Tebessi University, Constantine Road, Tebessa 12002, Algeria; ^3^Animal Biology and Physiology Research Laboratory (LBPA), Higher Teacher Training School of Kouba, Vieux Kouba BP 92, Algeria; ^4^Department of Chemical Engineering, School of Chemical Engineering and Mineral Industries, University of Ngaoundere, Ngaoundere 454, Cameroon

**Keywords:** anticancer activity, cigarette smoke, essential oils, *in vivo* oxidative parameters, *Juniperus oxycedrus*, *Juniperus phoenicea* L., toxicants

## Abstract

Tobacco smoke contains toxic chemical substances that mediate the generation of reactive oxygen species and lung cancer. This study investigates the potential of essential oils (EOs) from *Juniperus oxycedrus* (JOX) and *Juniperus phoenicea* L. (JPH) in reducing the harmful effects of cigarette smoke. The EO of JOX (JOX-EO) and JPH (JPH-EO) showed good inhibition of nicotine, with IC_50_ values of 16.78 ± 1.04 μg/mL and 18.40 ± 0.46 μg/mL, respectively. Moreover, JOX-EO had the greatest percentage reduction of basic tar (64.45%), followed by neutral tar (53.23%), and acidic tar (25.15%). Furthermore, the evaluated JOX-EO demonstrated efficacy in inhibiting polycyclic aromatic hydrocarbons (PAHs). In vivo experiments on rats chronically exposed to cigarette smoke reveal promising results regarding oxidative stress markers. The administration of JOX-EO at 200 mg/kg for 15 days postsmoking cessation shows significant improvements in hematological variables (red blood cells [RBC], platelets [PLT], hemoglobin [HB], hematocrit [HCT], and mean corpuscular volume [MCV]). The oxidative stress markers, including glutathione (GSH), malondialdehyde (MDA), glutathione S-transferase (GST), glutathione peroxidase (GPx), and catalase (CAT), were significantly reduced, indicating powerful antioxidant potential. The EOs exhibited concentration-dependent percentage inhibition of human lung carcinoma (A549) cell viability, with IC_50_ values indicating greater potential than etoposide, the standard used for comparison. Both EOs demonstrate the capacity to mitigate the lingering effects of smoking, such as oxidative stress and lung cancer, providing reassurance and comfort to those affected.

## 1. Introduction

The most widespread form of tobacco consumption, commonly referred to as smoking, is a major public health problem for both smokers and nonsmokers exposed to cigarette smoke. According to the World Health Organization (WHO), this major problem causes more than 8 million deaths each year, resulting from acute respiratory conditions and pulmonary diseases, cardiovascular disorders, periodontal diseases, and various types of cancers [1–5]. The cause of all these disorders is that cigarette smoke contains over 7000 chemicals, of which 86 are carcinogenic and cause oxidative stress [6, 7]. A variety of pathological disorders, including chronic bronchitis, emphysema, mucus hypersecretion, lung inflammation, chronic airflow obstruction, coughing, fatigue, and dyspnea, can result from exposure to cigarette smoke and its constituents [8]. It is important to recall that when oxidative stress becomes chronic, it is responsible for the majority of the diseases mentioned above. Active and inactive smoking can induce inflammatory and oxidative stress responses in the subjects [9]. Exposure to smoke is regarded as significant environmental risk factor for human health. It is linked to the development of lung cancer and several respiratory disorders, including chronic obstructive pulmonary disease [10]. Cigarette smoking explains almost 90% of lung cancer risk in men and 70%–80% in women, and it accounts for the production of reactive oxygen species (ROS) in living systems, leading to oxidative stress [11, 12]. Oxidative stress acts as a DNA-damaging agent, effectively increasing the rate of mutation within cells, which causes disruption in cellular division and thus promotes oncogenesis [13]. In addition, generated ROS resulting from smoking and other agents can activate specific signaling pathways, causing tumor development and cancer through the regulation of angiogenesis, cellular proliferation, and metastasis [14]. Cancers resulting from cigarette smoking are responsible for 85% of lung cancers and approximately one-third of all cancer deaths. However, they are easily preventable and can be dramatically reduced with tobacco cessation. All individuals have the power to reduce the risks of smoking and take responsibility for their health [15]. Reducing the number of cigarettes smoked can create a decline in the risks for lung cancer, but quitting completely remains the most effective way for people who smoke to reduce their risk. Furthermore, there is the issue of chronic addiction treatment, and even when a smoker feels the need to quit smoking, it takes years for his body to detoxify. Psychological side effects accompany this. Reducing or avoiding exposure to cigarette smoke offers a clear chance to stop diseases linked to it. Even though studies assessing the impact of exposure to tobacco smoke generally resemble toxicological experimental settings, there is currently no particular framework that evaluates the risk associated with cigarette smoke exposure and means of reducing or stopping it [16]. It should be pointed out that even with the use of certain pharmaceutical drugs, the toxicity of a hundred harmful substances cannot be reduced because these medicines contain only one active substance.

The development of substances that can reduce one or more tobacco toxicants and their human exposure is greatly encouraged, as it will reduce the risk of cigarette smoke–related diseases and their adverse health effects [17]. Some methods have been used to reduce the toxic substances in smoke. The use of filter adsorbents, treatment or dilution of tobacco, the development of cigarette papers with high permeability, increased filtration efficiency, and filter ventilation are among the methods used to reduce some toxicants in the smoke phase [17, 18]. Certain materials, such as activated carbon, graphene, zeolite, cellulose, and specific polymers, can help to reduce the levels of major toxicants in cigarette smoke, including nicotine, carbon monoxide, polycyclic aromatic hydrocarbons (PAHs), hydrogen cyanide, tobacco-specific nitrosamines, and formaldehyde [6, 19]. As reported in the literature, many studies have been conducted to evaluate the potential benefits of different plant-derived naturals, such as natural antioxidants [20]. Juniper essential oils (EOs), such as the EO of *Juniperus communis* L., *Juniperus sabina* L., and *Juniperus sibirica*, have unique properties that make them effective in reducing harmful cigarette smoke toxicants, such as their high antioxidant content and ability to neutralize free radicals [21, 22].

In a significant step toward enhancing the value of native juniper plants in Algeria, this study investigated the potential of EOs from *Juniperus phoenicea* L. (JPH) and *Juniperus oxycedrus* (JOX) in reducing some harmful chemicals in cigarette smoke. The major target toxicants in this study are nicotine, tar, and PAHs. Furthermore, the study evaluated the effects of the EOs on oxidative stress parameters and human lung adenocarcinoma A549 cell lines, offering a hopeful perspective for the future of tobacco harm reduction.

## 2. Materials and Methods

### 2.1. Plant Material

In December 2018, berries of JOX and JPH were collected from the Doukane area in Tebessa, Algeria (35°19′20″ N). Soraya Hioun from the Laboratory of Applied Chemistry and Renewable Energies (LACRE) at Echahid Cheikh Larbi Tebessi University conducted a thorough identification and authenticated them as wild shrubs belonging to these two species [23]. This unique location provided us with valuable specimens for our research.

### 2.2. Extraction of EOs

The EOs were extracted using the hydrodistillation technique with a Clevenger-type apparatus. The plant material-to-water ratio was 2:10 (w/v), with 300 g of fresh, crushed berries added to a two-liter round-bottom flask containing 1500 mL of distilled water. The flask was then placed on a heating mantle for 3 h. Vapors loaded with EOs were passed through a 50-cm vertical tube and then into the cooler, where they condensed into droplets that were carefully collected in a separating funnel alongside water molecules. The two phases formed were separated, and the oil phase was dried using anhydrous CaCl_2_. The recovered oil, slightly yellow in color, from JOX (*Juniperus oxycedrus* essential oil [JOX-EO]), and JPH (*Juniperus phoenicea* essential oil [JPH-EO]) was stored in tightly closed brown EO bottles and conserved in a freezer at 4°C pending assays.

### 2.3. Nicotine Isolation and Measurement

Ten gram of tobacco (of RYM cigarettes, an Algerian production) and 200 mL of a 30% aqueous sodium hydroxide solution were mixed in a 500-mL flask. The mixture was then subjected to a systematic rotary evaporator distillation under reduced pressure (100 mbar) and temperature (70°C). The distilled solution was recovered after 20 min of condensation, and the nicotine was extracted using three successive liquid–liquid extractions with 25 mL of dichloromethane (DCM) each. A small amount of anhydrous sodium sulfate was added to dry the organic phase before filtration. The filtrate was then evaporated under vacuum to afford a yellowish nicotine oil.

#### 2.3.1. Sample Preparation

The nicotine extract stock Solution (20 μg/mL) A was prepared by dissolving 2 mg of nicotine in distilled water and adjusting the volume to 100 mL. Solution B with a concentration of 12 μg/mL was produced from this solution. Stock solutions (C1 and C2) were prepared by dissolving 2 μL of EOs (JOX-EO and JPH-EO) in 100 mL of acetone solution. The EO dilutions were prepared from stock solutions (C1 and C2) and diluted to final concentrations ranging from 3.8 to 19 μg/mL. These solutions, along with Solution B, were combined in varying ratios (v:v), and thoroughly mixed. They were then left at room temperature for a crucial 15 min, a period of time that is significant in allowing the solutions to equilibrate, before measuring the nicotine concentration.

#### 2.3.2. Nicotine Dosage by UV Spectrophotometry

The method of Al-Tamrah [24] was adopted for the dosage of nicotine with slight modifications. A 5-mL test tube was introduced with exactly 200 μL of potassium permanganate (2.08 × 10^−3^ M), followed by 400 μL of sodium hydroxide (5 M). The resulting mixture was then swirled gently to homogenize. To this mixture, 3.4 mL of each sample, the standard, or the blank was added. That is, 3.4-mL volume of Dx solutions (solutions of nicotine with the EOs solutions at different concentrations), the standard (Solution B/acetonitrile, v/v), or the blank (acetonitrile/distilled water, v/v). The sample preparation was meticulous, ensuring the reliability of the experiment. The solution was made up to a volume of 5 mL with distilled water, then shaken well, and the absorbance was measured at 610 nm after 180 s.

A calibration curve using pure nicotine was established under the same conditions as the test samples, with different concentration ranges between 2 and 20 μg/mL. The following formula expresses the ability to reduce or inhibit nicotine:

(1) $$\begin{array}{c} \text{Inhibition }\%=\frac{\text{Abs }C-\left(\text{Abs }S-\text{Abs }E\right)}{\text{Abs }C}\times 100, \end{array}$$

where Abs C is the absorption of the control (the nicotine solution without the EO), Abs E is the solution absorption when just the EO is present, and Abs S is the amount of nicotine solution that is absorbed when the EO is present.

Experiments were carried out in triplicate, and the IC_50_ value was measured as the substrate concentration that causes the loss of 50% of the activity of the nicotine.

### 2.4. Effect of Investigated EOs on Different Tars Contained in Cigarette Smoke

The cigarette smoke collection technique was used in obtaining the tar fractions found in the mainstream smoke of cigarettes. These tar fractions are treated with both EOs (JOX-EO and JPH-EO), and then quantitatively assessed for the EOs' effect on the neutral, acidic, and basic tar fractions.

#### 2.4.1. Cigarette Smoke Collection

Benzene was used to extract the chemicals contained in cigarette smoke, such as nicotine, tar, and carbonyl compounds. To maintain flexibility in adjusting the cigarette smoking parameters, the mainstream smoke of RYM cigarettes (an Algerian brand) was generated at a rate of seven puffs per cigarette by smoking of the cigarettes according to the puffing regime parameters of the CORESTA approach [25], with minor modification. Aspirations lasted 2-3 s of an interval of 1 puff/30 s with a volume puff of 50 m^3^. In 150 mL of benzene, bubbles were introduced with an equivalent of 644 puffs, or 90 cigarettes' worth of smoke. This process, in accordance with the principles of a cigarette's pyrolysis and combustion, it was able to recover the majority of the chemicals found in cigarette smoke, demonstrating the effectiveness of the method.

#### 2.4.2. Extraction of Tar Fractions From Benzene Extracts of Cigarette Smoke (BECS)

**Figure 1:**
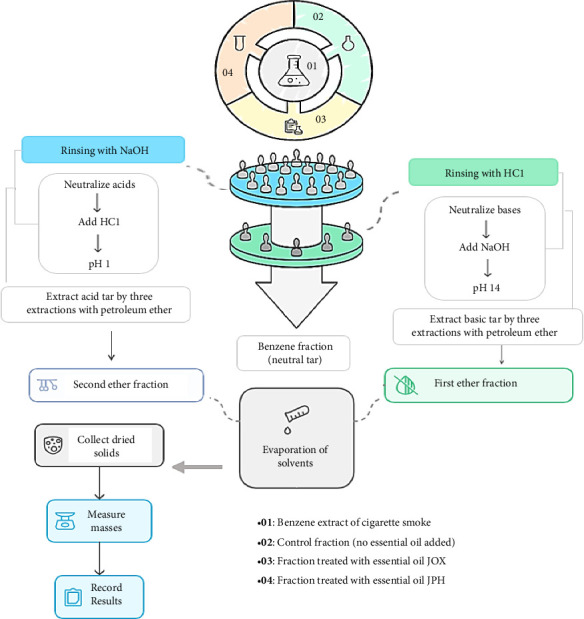
Experimental procedure for the extraction of tar fractions from the benzene extract of cigarette smoke (BECS).

To evaluate the effectiveness of JOX and JPH EOs in reducing tar rates, with utmost precision, the BECS from 90 cigarettes was divided into three equal parts: one control and two treated with different EOs (JOX-EO and JPH-EO). In each treated solution, 100 μL of EO was added to 50 mL of the benzene solution or recovered cigarette smoke extract (RCSE), followed by vigorous mixing. The solutions were incubated in the dark at room temperature for 18 h. After incubation, the solutions were rinsed with NaOH (1 N) to extract acid tar and then with HCl (1 N) to extract alkaline tar. The pH of the resulting acid and basic solutions was adjusted using concentrated NaOH and HCl until pH levels of 14 and 1 were achieved, respectively. Acid and basic tars were extracted through three extractions with petroleum ether for each solution, yielding three fractions: the benzene fraction (neutral), the first ether fraction (basic), and the second ether fraction (acid). These fractions were evaporated under vacuum using a rotary evaporator to remove solvents, and the solids were dried at 25°C for several hours before their masses were measured in milligrams (mg). The procedure is summarized in Figure 1.

### 2.5. PAHs Extraction and Characterization

#### 2.5.1. PAHs Liquid–Liquid Extraction

One hundred and fifty cigarettes' worth of smoke was extracted using a solution of hexane/ethanol (2:1). In a separating funnel, 50 mL of distilled water was added to a volume of 150 mL of the organic solution of cigarette-derived smoke extract, and the organic and aqueous phases were separated after stirring. The lower phase was then meticulously washed three times with 25 mL of hexane, ensuring the purity of the collected PAHs. The hexane phases containing the PAHs were then collected and evaporated at 40°C.

#### 2.5.2. Ensuring the Purity of PAHs

Column chromatography was used to purify the resultant PAHs. A slurry of 20 g of silica and 250 mL of a hexane/DCM (1/1) (v/v) mixture was introduced into a 2-cm-diameter column. Two hundred and fifty milliliter of a hexane-DCM mixture was used as the mobile phase and eluted at a flow rate of 30 mL for 25 min. Then, 8-mL fractions were recovered with great care in small, labeled brown glass vials, ensuring the integrity of the samples. The obtained fractions were analyzed using UV spectroscopy.

#### 2.5.3. Pretreatment of PAHs With EO

All collected PAH fractions were divided into two parts: one served as a control and the other was treated with the EO. In each fraction (4 mL), a volume of 2.5 mL of JOX-EO is added. This mixture is then stirred for 15 min in the dark, a crucial step to ensure thorough mixing and interaction. After this, the mixture will be analyzed by UV-Vis spectrophotometry.

#### 2.5.4. Spectrophotometric Analysis

A Shimadzu UV-1700 Pharma-Spec spectrophotometer was used for the UV analysis, with 10-mm quartz measuring cells, before and after treatment with JOX-EO (dissolved in hexane-DCM 1:1 [v/v]). The hexane-DCM 1:1 (v/v) was also used as a blank. The recorded spectra of each PAH fraction were taken between 200 and 400 nm.

### 2.6. Evaluation of In Vivo Oxidative Parameters

#### 2.6.1. Animals

Adult Wistar rats (*Rattus norvegicus*) weighing between 86 and 133 g were provided from the Pasteur Institute of Algiers (Algeria). Rats were housed in a conditioned room maintained at a temperature of 23 ± 2°C, with 60% humidity, hygrometry, and under a 12-h light/dark cycle, with adequate provision of food and water under strict hygiene conditions. The rats were housed in six groups, each consisting of four rats per standard Makrolon cage. Following the requirements of the European Council Directive (EU2010/63) [26] on the care of laboratory animals, every effort was taken to decrease animal suffering and the number of animals used in the research.

#### 2.6.2. Cigarette Smoke Exposure

The animal's tobacco smoke exposure setting follows the principle outlined in the literature [27, 28] with slight modifications. The rats were kept in Plexiglas cages (50 × 30 × 30 cm) with wooden bedding and exposed to cigarette smoke. Throughout the tobacco smoke exposure sessions, the rats (full body exposure) were not confined. The smoker group was exposed to the smoke of four filtered commercial cigarettes each day for 1 month in a unique device made to expose the rats to cigarette smoke for 2 hours. Tobacco smoke was produced by burning filtered cigarettes; once the cigarette was lit, the suction was activated using a standardized smoking procedure (35 cm^3^ puff volume, 1 puff per minute, 2 s per puff). The rats were moved to the exposure cages just before the tobacco smoke exposure session and then placed back in their original cages.

#### 2.6.3. Sample Administration

Rats were divided into six groups, each containing four rats. The animals were randomly divided into control and experimental groups. A control group (C) received distilled water, a COX and CPH control group were treated with JOX-EO and JPH-EO (200 mg/kg each), a CS smoking-withdrawn group stopped being exposed to cigarette smoke after one month, and a STOX and STPH smoking-withdrawn groups, a unique and complex combination, stopped being exposed to cigarette smoke and were administered JOX-EO and JPH-EO (200 mg/kg each), respectively.

#### 2.6.4. Rats and Their Relative Organs' Weight

Throughout the experimental study, rats' bodies were weighed every 2 days, and the relative organ weights were determined using the following formula:

(2) $$\begin{array}{c} \text{Relative organ weight }\left(\%\right)=\frac{\text{absolute organ weight }\left(g\right)}{\text{body weight }\left(g\right)}\times 100. \end{array}$$

#### 2.6.5. Hematological Analysis

Using the automated hematology analyzer (Mindray, BC-31s), the following hematological parameters were measured: red blood cells (RBCs), platelets (PLTs), hemoglobin (HB), hematocrit (HCT), and mean corpuscular volume (MCV).

#### 2.6.6. Dosage of Oxidative Stress Biomarkers

For each group, lung and brain samples were used to measure enzymatic and nonenzymatic biomarkers of oxidative stress, such as glutathione (GSH), malondialdehyde (MDA), glutathione-S-transferase (GST), glutathione peroxidase (GPx), and catalase (CAT). A sample of 500 mg of tissue is used for the assay. After the deproteinization with 0.25% sulfosalicylic acid, the GSH was determined using the method of Esterbauer et al. 1992 [29], and the MDA was determined using the method of Weckbecker and Cory 1988 [30]. In terms of enzymatic parameters, GST was found using the method from Habig et al. (1974) [31], GPx using the method from Flohé and Gunzler (1984) [32], and CAT using the method from Cakmak et al. (1991) [33].

### 2.7. Anticancer Activity of EOs Against Lung Cancer Cells

A549 cell lines for human lung cancer were obtained from the American Type Culture Collection (ATCC). The 3-(4,5-dimethylthiazol-2-yl)-2,5-diphenyltetrazolium bromide (MTT) assay colorimetric cell viability method was applied in determining the cytotoxicity of the EOs against small lung cancer A549 cell lines, as described previously [34]. The cells were cultured in Dulbecco's Modified Eagle Medium supplemented with 10% of heat-inactivated fetal bovine serum, kanamycin (100 μg/mL) and amphotericin B (5.6 μg/mL) and incubated overnight. Into each well of a 96-well microplate was introduced 1-μL aliquots of EOs solutions (16–0.25 mg/mL in DMSO) in 99 μL of media containing 5 × 10^3^ lung cancer cells. Wells without cancer cells served as the blank. The plate was incubated under 5% CO_2_ atmosphere at 37°C for 72 h. The medium was pipetted out and 100 μL of MTT solution (0.5 mg/mL) were introduced into each well followed by further incubation for 1.5 h. About 100 μL of DMSO was added to each well to dissolve the MTT reduction product (formazan crystals). The absorbance of each well was measured using a spectrophotometer (Thermo Scientific Multiskan FC, Vantaa, Finland) at 540 nm. Etoposide served as a positive control, while DMSO was used as the negative control. Then, 1 μL of each dilution solution of etoposide (16–0.25 mg/mL in DMSO) were introduced to the corresponding wells with 99 μL of media. Final EO and etoposide serial concentrations ranged from 160 to 02.5 μg/mL. All measurements were done in triplicate and results reported as the mean ± SE of three parallel experiments. The percentage inhibition was calculated as given below.

(3) $$\begin{array}{c} \%\text{ inhibition of cell viability}=\left[1-\frac{\left(A_{\text{test}}-A_{\text{blank}}\right)}{\left(A_{\text{control}}-A_{\text{blank}}\right)}\right]\times 100, \end{array}$$

where *A*_control_ is the absorbance of control (DMSO), *A*_test_is the absorbance of the test sample, and A_blank_ is the absorbance of the cell-free wells.

Cytotoxicity was expressed as the concentration of oil inhibiting cell viability by 50% (IC_50_).

### 2.8. Statistical Study

GraphPad Prism 8 was used to calculate the mean ± SD for four measurements, and the Holm–Sidak method with an alpha level of 0.05 was used to see how statistically significant the differences were between the treated groups and the controls. Without assuming a constant SD, each row was examined independently, and to better visualize the data, use the Microsoft Excel 2010 to create graphs and histograms.

## 3. Results

### 3.1. EO Yield

According to Table 1, the distillation time needed to collect the maximum EO by the hydrodistillation technique was 180 min.

### 3.2. Nicotine Reduction

Following the steps outlined in the previous section for extracting nicotine, 20 mg of nicotine was recovered. This was confirmed by the appearance of a greenish-yellow color on the TLC plate after adding potassium permanganate, a crucial step in the identification process as it gives nicotine its organoleptic properties [4]. Nicotine is a colorless to pale yellow, oily liquid that absorbs water.

**Figure 2:**
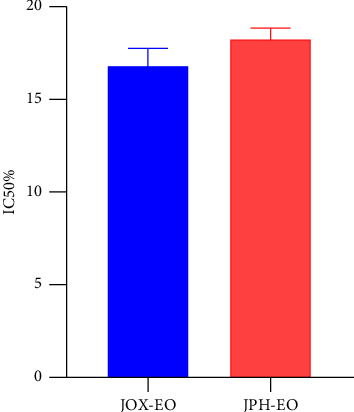
IC_50_% of nicotine treated by EOs (JOX = 16.78 ± 1.04, JPH = 18.40 ± 0.46).

**Figure 3:**
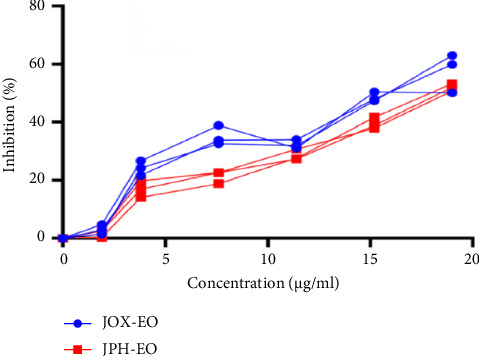
Inhibition percentage of nicotine by JOX-EO and JPH-EO.

**Figure 4:**
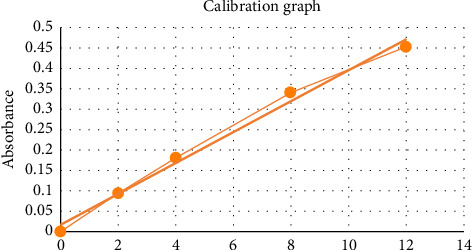
Nicotine concentration calibration graph (Absorbance = 0.0379 [nicotine] + 0.0166), with a correlation factor *R*^2^ = 0.991 and concentrations in μg/mL.

**Figure 5:**
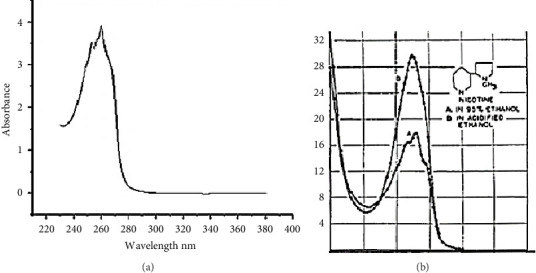
Nicotine spectrum: (a) represents the extracted nicotine spectrum; (b) represents the pure nicotine spectrum (in ethanol, 95%).

The obtained results were carried out in triplicate, and the mean and standard deviations (SD) are shown in Table 2. The concentration of nicotine (μg/mL) is determined by reference to the calibration graph.  Figure 2 shows IC_50_% of nicotine treated by EOs.  Figure 3 indicates the inhibition percentage of nicotine by JOX-EO and JPH-EO, while Figure 4 shows the nicotine concentration calibration graph. The UV spectrum of the extracted nicotine and that of pure nicotine samples are presented in Figure 5.

### 3.3. Tar Reduction

The findings of the weight decrease of the neutral, alkaline, and acidic tar fractions after the addition of the investigated EOs and the controls used to treat tar-containing benzene solutions are presented in Table 3.

**Figure 6:**
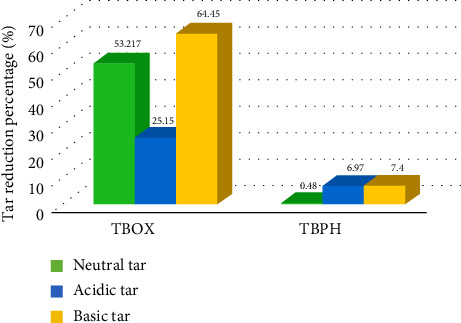
Tar reduction percentage via the investigated essential oils (NT: neutral tar; AT: acidic tar; BT: basic tar).

Figure 6 illustrates the significant tar-reducing potential of the EOs of JOX which has a significant tar-reducing power. As can be noted, even from a volume of 100 μL, the EO of JOX berries presented a high and significant tar-inhibiting power for the three fractions of tar: neutral, acidic, and basic, respectively, with percentages of inhibition of 69.11%, 62.86%, and 84.57%, respectively. These data support the potential for these EOs to be used in therapeutic or industrial applications. However, the berries JPH-EO had an inhibition percentage less than 10% (0.50%–7.70%) for all tar fractions, indicating that their inhibitory powers for 100 μL cannot be conclusively determined at this stage.

### 3.4. PAHs

**Figure 7:**
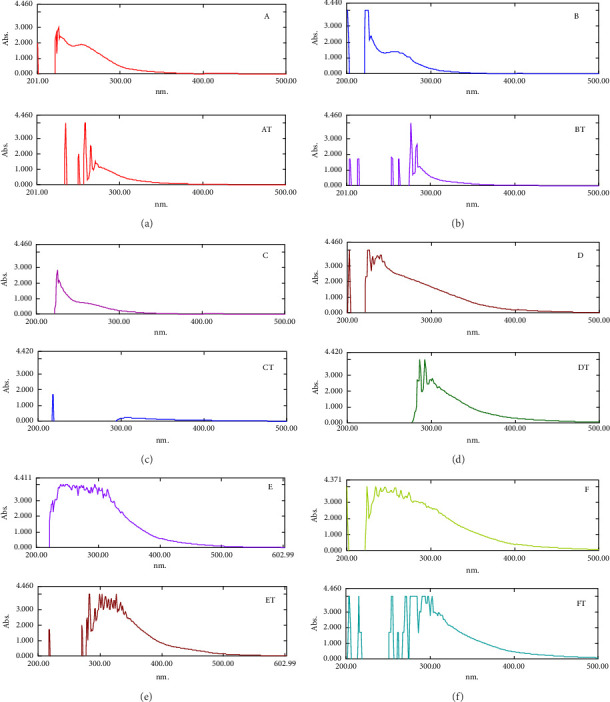
Spectra of solutions containing PAHs treated with JOX-EO with their control solutions (control fractions: (a, b, c, d, e, and f). Treated fractions with JOX-EO are: AT, BT, CT, DT, ET, and FT).

After the evolution of the chromatographic column monitored by TLC, six fractions were collected (A, B, C, D, E, and F). From the solutions treated with JOX-EO and analyzed by UV spectrophotometry, the PAHs' UV spectra were obtained for each standard or treated solution. The spectra are illustrated in Figure 7.

### 3.5. Body and Relative Organ Weights

Table 4 gathers the calculated relative organ weight (brain and lung bodyweight ratios) of the smoking-withdrawn rats. These rats were treated with JOX- and JPH-EOs for 2 weeks, following a precise and rigorous experimental methodology.

### 3.6. Hematological Analysis

Table 5 shows the hematological parameters (RBC, PLT, HB, HCT, and MCV) of Wistar rats that were given JOX-EO and JPH-EO by oral administration for 2 weeks. All parameters hematologically tested were compared with those of the control rats.

### 3.7. Measurement of Oxidative Stress Biomarkers

**Figure 8:**
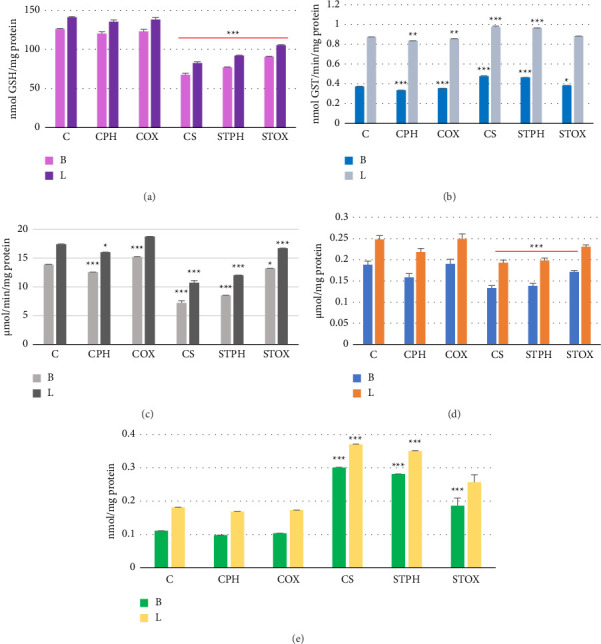
Intracellular levels of oxidative stress markers: (a) glutathione-S-transferase GST, (b) glutathione GSH, (c) glutathione peroxidase GPx, (d) catalase CAT, and (e) malondialdehyde (MDA) in the lung (L) and brain (B) following 2 weeks of treatment by the essential oils in smoking cessation rats relative to the control group; ^∗^(*p* ≤ 0.05); ^∗∗^(*p* ≤ 0.001); ^∗∗∗^(*p* ≤ 0.0001).

Figures 8(a), 8(b), 8(c), 8(d), and 8(e) illustrate the intracellular levels of oxidative stress biomarkers (GST, GSH, GPx, CAT, and MDA) in the lung and brain after 2 weeks of treatment in smoking-withdrawn rats compared to the control group. According to the results presented in Figure 8(a), there were no significant changes in GSH levels (*p* > 0.05) in the control groups (CPH and COX) treated with EOs, indicating that JPH-EO and JOX-EO do not account for a consistent increase in GSH levels under healthy conditions. However, there was a significant decrease (*p* < 0.0001) in the smoking-withdrawn group (CS) and the smoking-withdrawn group treated with JPH-EO and JOX-EO (STPH and STOX), with different percentages of GSH decrease observed for the CS (46.33%), STPH (38.83%), and STOX (28.20%) groups compared to the control group. For the other parameters in Figure 8(b), the GST levels decreased in the CPH (*p* < 0.0001) and COX (*p* = 0.001) groups but significantly increased (*p* < 0.0001) in the CS and STPH groups. Nevertheless, remarkably, STOX exhibited GST levels close to those of the nonsmoking group.  Figure 8(c) indicates significant changes in lung and brain CAT levels across various experimental groups. In the smoking-withdrawn control group (CS), the smoking-withdrawn group treated with JPH-EO (STPH), and the control group treated with JPH-EO (CPH), CAT levels decreased significantly (*p* < 0.0001), reflecting an increase in oxidative stress. Conversely, the control group treated with JOX-EO (COX) showed a notable increase in CAT levels (*p* < 0.0001), suggesting a protective effect against oxidative damage. The smoking-withdrawn group treated with the JOX-EO (STOX) group demonstrated CAT levels closer to the control group than to the smoker group. The results indicated a significant increase (*p* < 0.0001) in the CAT level, indicating that JOX-EO may effectively mitigate oxidative stress during withdrawal.  Figure 8(d) indicates that, in the CPH, COX, and STOX groups, the levels of GPx did not change (*p* > 0.05) and were similar to those in the nonsmoking control group (C). However, the smoking-withdrawn control group (CS) and smoking-withdrawn group treated with JPH-EO (STPH) showed a decrease in the levels of GPx. Also, in Figure 8(e), the MDA levels in the lungs and brain showed a significant decrease in the CPH and COX groups (*p* < 0.0001). Conversely, the CS and STPH groups exhibited significantly higher MDA levels (*p* < 0.0001). The STOX group had increased MDA levels (*p* = 0.02), which were relatively similar to those of the control group (C). Notably, when compared to the CS group, the decrease in MDA levels was also significant (*p* = 0.0025). Hence, the observed increase in GST and MDA indicates an adaptive response of the body to exposure to toxins from cigarette smoke, highlighting the body's resilience and the importance of further research into these adaptive mechanisms.

### 3.8. Anticancer Activity of EOs

**Figure 9:**
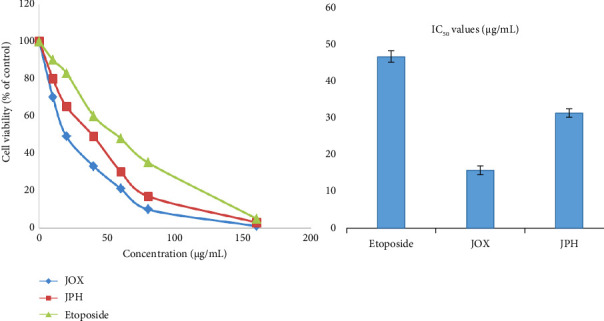
Cell viability inhibition and cytotoxicity of the EOs against human lung adenocarcinoma cell lines (A549): *J. oxycedrus* (JOX) and *J. phoenicea* (JPH) essential oils.

Smoking cigarettes is a major risk factor for lung cancer since some of the cigarette smoke components are carcinogenic. The potential cytotoxicity of the EOs from berries of JOX and JPH against human lung adenocarcinoma cell lines (A549) was evaluated and reported as % cell viability with respect to control and IC_50_ in Figure 9. The cytotoxic activities of the EOs on the A549 lung cancer cell lines, after treatment with serial concentrations of EOs for 24 h, were evaluated using the MTT viability assay. The EO exhibited concentration-dependent decrease in cell viability and exhibited IC_50_ values of 15.88 ± 1.25 μg/mL (JOX) and 31.5 ± 1.11 μg/mL (JPH) against the human lung adenocarcinoma cell lines (A549), as shown in Figure 9. Importantly, the results indicate that the EOs were more active than the standard compound etoposide, which showed an IC_50_ value of 46.9 ± 1.55 μg/mL, a significant finding that underscores the potential of these EOs in cancer treatment.

Cell viability, a critical measure in cancer research, refers to the number of healthy cells within a cancer cell population. It is instrumental in evaluating the efficacy of cancer treatments, aiding in the determination of the medication's lethality. Notably, as the concentration of EOs increases, cell viability decreases. At equivalent concentrations, EOs demonstrate a higher activity level than etoposide, a significant finding. Moreover, JOX-EO exhibits a higher activity level than JPH-EO.

## 4. Discussion

The purpose of the current study was to assess the ability of EOs from two juniper species to reduce harmful substances found in cigarette smoke to evaluate the in vivo oxidative status of rats exposed to cigarette smoke, as well as the cytotoxicity of the EOs against lung cancer. The results give a global significance of both EOs in mitigating the adverse effects of cigarette smoking on human health. The characteristic wavelength (*λ*_max_) of nicotine, which is 260 nm, is a key parameter in our study. This wavelength can be used to detect the presence of nicotine in samples through the UV spectrum. However, the obtained spectrum of the extracted nicotine (Figure 5(a)) was compared with that of pure nicotine used as a reference (Figure 5(b)) [35]. From the obtained results, it can be seen that both spectra have similar characteristic peaks at the same *λ*_max_. Furthermore, in the UV-visible range, no additional peaks were observed, indicating that other impurities are not present in the obtained extract. Nevertheless, it is not possible to say that only (S)-(-)-Nicotine is obtained because tobacco contains three different isomers of nicotine, including (S)-(-)-Nicotine, (R)-(+)-Nicotine, and the analog nor-nicotine [36]. (S)-(-)-Nicotine is reported to be the most abundant compound in tobacco [36]. Tamrah's principle, with little modifications, was used to measure the nicotine content. To evaluate the nicotine content, the sample should be reacted with potassium permanganate in the presence of sodium hydroxide to generate a green product as a result of the permanganate reduction to manganate [24]. In the presence of EOs, nicotine is insoluble, and so acetonitrile was added to ease dissolution and assess the reduction of nicotine levels upon treatment with EOs. According to Figures 2 and 3, both EOs (JOX-EO and JPH-EO) showed a significant decrease in nicotine. JOX-EO (IC_50_ = 16.782 ± 1.043 μg/mL) exhibited more nicotine inhibition compared to JPH-EO (IC_50_ = 18.405 ± 0.463 μg/mL). When a cigarette burns, a high temperature is reached that triggers pyrolytic processes and the creation of radical species. Some of these radicals may interact with different substances found in cigarette smoke, forming new substances [37]. According to the shortlist of harmful and potentially harmful constituents published by the FDA, constituents from a variety of chemical classes (such as PAHs and tobacco-specific nitrosamines are involved [38]. The majority of hazardous and cancer-causing components are contained in the tar, whose concentrations determine the toxicity of cigarette smoke. Weight loss results were used to deduce the effects of the EOs in reducing the tar (acidic, basic, and neutral tar) content. Acidic and basic tar fractions were both brown-colored, while the neutral tar fraction had a darker color. It is reported that the brown-colored tar is composed of polymerized substances of varying molecular weights resulting from the pyrolysis during cigarette burning [39]. The addition of JOX-EO decreased the intensity of the dark color of the neutral tar. On the other hand, no alteration of the tar color was noted upon addition of JPH-EO. Based on the obtained results, JOX-EO was more effective in reducing the tar content. JOX-EO had the highest tar inhibition percentage in neutral (53.23%), acidic (25.15%), and basic (64.45%) tar fractions.

The EOs were tested for their potential to interact with and inhibit PAHs. PAH spectra are higher homologs of benzene, so their appearance depends on the number of aromatic rings and their arrangement in the molecule, giving more structured spectra for heavier compounds [40, 41]. In general, the absorption range of the PAHs is between 200 and 460 nm [40]. The UV spectrum analysis of Fraction A reveals the existence of three chemicals, which aligns with the observations made during the TLC analysis. From the UV spectra (Figure 7(a)), three intense peaks are observed at 223 nm, 226 nm, and between 228 and 230 nm, confirming that these substances are light PAHs, in addition to a broad peak between 245 and 325 nm. TLC of Fraction B indicated two substances, and the spectrum (Figure 7(b)) shows that these substances contain at least one light PAH. It also exhibits two strong peaks, one at 224 nm and the other at 229 nm, as well as a broad peak between 245 and 330 nm. For Fraction C, the TLC showed a single spot, indicating that it is likely a single substance. The spectrum (Figure 7(c)) also shows that it is a light PAH, as evidenced by its strong peak at 224 nm and a broad peak between 250 and 300 nm. For other fractions, D contains a single spot, as distinguished from the TLC, so possibly a unique substance, while at least two substances are present in Fractions E and F. The spectra (Figures 7(d), 7(e), and 7(f)) of the last three fractions show that they are most likely heavy PAHs because they are more structured or stand out in the 225–400 nm range. After analyzing the spectra of the previously treated fractions with *JOX-EO* and superimposing them with those of the controls, differences were observed in the general appearance of the spectra, including chemical shifts and peak intensities. Moreover, in the near UV range between 200 and 300 nm, additional thin peaks of varying intensities appeared with a hyperchromic effect for Fractions A and B. It is reported that a hyperchromic effect is observed when aromatic rings are less condensed [41]. This suggests some transformation with the appearance of new chemicals aside from PAHs. For Fraction C, a hypsochrome effect was observed in the peaks (see Figure 7(c)) and the disappearance of the large band, which probably means that the PAH has been transformed into another product by degradation, accompanied by the appearance of a small peak that may indicate the presence of acyclic dienes. In Fraction D, a bathochromic effect was seen on the peaks (see Figure 7(d)). This could be because the number of rings and conjugations increased, or other substitutions appeared. Similarly, for Fractions E and F, a bathochromic effect was observed in certain peaks, with the appearance of other fine peaks in the region between 200 and 275 nm (Figures 7(e) and 7(f)). These peaks, which are more intense in the spectrum of Fraction F, indicate the transformation of heavy PAHs with the formation of other products. It can therefore be concluded from the shape of the obtained spectra that there is an interaction between the PAHs and JOX-EO. This interaction may generate other products that differ from the PAHs or the degradation of the latter. However, the identification of these new products will require the use of additional high-performance separation and identification techniques.

Many studies showed that the toxic components of cigarette smoke can have various effects on different organ systems, and smoking has been associated with an increased risk of the development of atherosclerosis, polycythemia, obstructive pulmonary disease, cardiovascular diseases, and myocardial infarction [1, 42–45]. This is evident in some reports about the hematological components of smokers and nonsmokers, which demonstrated that smoking increases the amounts of RBC, HB, HCT, MCV, mean corpuscular hemoglobin (MCH), and white blood cells (WBC) [44, 45]. In the present study, comparing rats exposed to cigarettes and those not exposed, it is observed that smoking increases the counts of RBCs and PLTs. Still, the other components, including HB, HCT, and MCV, decreased. The decrease in HB, HCT, and MCV counts could be attributed to lower carbon monoxide consumption during smoking cessation during the 15-day study period. HCT and MCV counts increased in response to anoxia due to carboxyhemoglobin generated during smoking [46]. MCV measures the size of a RBC, and the presence of RBCs that are smaller or larger than normal size indicates anemia [45]. Low MCV levels were observed in the analysis of COX (control treated with JOX-EO), CS (control exposed to smoke), and STPH (smoking group treated with JPH-EO) groups, which may imply microcytic anemia. Lower-than-normal HB levels result from illnesses such as iron deficiency or lead poisoning [47]. These findings demonstrate that JPH-EO had no significant beneficial influence on reducing the hematological system risk associated with smoking. Hematological analyses (Table 5) of the Wistar rats group (CPH) treated with JPH-EO for 2 weeks through oral administration, compared with the nonsmoker group (C), indicate a significant decrease (*p* < 0.005) in RBC and PLT but had no effects on HB, HCT, and MCV (*p* > 0.05), while RBC, PLT, HB, and MCV were significantly (*p* < 0.05) decreased in the group (COX) treated with the JOX-EO. On the contrary, the withdrawing smoking group (CS) indicated a very significant increase (*p* < 0.005) in RBC and PLT and a significant decrease (*p* < 0.005) in HB and MCV but had no important effects on HCT. Moreover, the same remark was noted for the smoking-withdrawn group treated with JPH-EO (STPH) indicating that this biological response may be related only to the antioxidant effects of JOX-EO.

Antioxidant defense systems are found in various tissues, including the lungs and brain, where they protect cells from damage caused by ROS. Enzymatic antioxidants, such as superoxide dismutase (SOD), GPx, and CAT, can neutralize radicals through their sequential action, converting them into nontoxic species including water (H_2_O) and oxygen (O_2_) [48, 49]. The GSTs and cytochromes P450 (CYPs) play a role in the removal of ROS and the detoxification of cytotoxic compounds [50]. On the other hand, a significant nonenzymatic antioxidant is GSH, which acts as a cofactor for several enzymes that reduce oxidative stress; for example, it acts as an electron donor and is oxidized to GSH disulfide [51]. Glutathione reductase (GR) is capable of reducing the GSH disulfide back to GSH [52]. This process enables the body to eliminate ROS and keep redox equilibrium in a healthy state. However, the imbalance between free radicals and antioxidants, specifically the increase in free radicals in the body, can lead to oxidative stress. Subsequently, a chemical process known as lipid peroxidation produces MDA, which is accompanied by cell damage and contributes to various health issues [45]. Cigarette smoke is among the most important causes of oxidative stress and antioxidant loss because it contains powerful oxidants and substances that generate ROS that destroy biomolecules such as lipids, proteins, and DNA [7]. Moreover, ROS are created by endogenous oxidative species as they activate redox-sensitive signaling pathways to alter cellular responses [52]. Additionally, volatile organic compounds such as nicotine and PAHs can increase the formation of ROS [7]. Inhibition or reduction of the content of nicotine and PAHs in cigarette smoke can mitigate the oxidative stress induced by tobacco smoking. Cigarette smoke causes oxidative stress, and upon treatment with the EOs, in vivo antioxidant status was improved in rat models. In this study, we measured the levels of oxidative stress indicators (GPx, CAT, GST, GSH, and MDA) in the lung and brain tissues of rats previously exposed to cigarette smoke, following treatment with EOs. The EOs displayed efficacy in decreasing oxidative stress. Several studies on the acute effects of cigarette smoking on oxidative stress in humans and animals have revealed that smokers have lower levels of GSH in their lung cells, which may contribute to the onset of lung illness [53, 54]. The observed reductions in GSH levels in the smoking-withdrawn group treated with EOs could suggest a complex interaction where EOs might help mitigate some oxidative stress but not sufficiently restore GSH levels to normal levels. This indicates that JOX-EO plays a role in modulating oxidative stress, potentially preventing or mitigating damage rather than increasing endogenous antioxidants such as GSH. Their antioxidant effects may be more pronounced under oxidative stress conditions. The observed decrease in GST levels following treatment with EOs does not necessarily indicate the presence of oxidative stress. Instead, it may suggest that the EOs interfere with the body's detoxification processes or alter the metabolic pathways involved in GST production. In contrast, the significant increase in GST levels in the smoking-withdrawn group indicates a compensatory response by the rats' metabolism, stimulating the production of GST to enhance detoxification and protect against the oxidative stress typically associated with smoking cessation. This adaptive mechanism highlights the body's effort to restore homeostasis after exposure to harmful substances found in tobacco smoke. An increase in GST levels can occur as a compensatory response to oxidative stress caused by environmental factors such as smoking. When the body encounters increased levels of ROS or harmful substances, GST expression may rise to enhance detoxification processes and protect cells from damage [55, 56]. In this study, GST levels in the smoking-withdrawn group treated with JOX-EO (STOX) were closer to those of the control group (C), suggesting that JOX-EO may effectively support the restoration of antioxidant defenses and facilitate detoxification processes, potentially providing a protective effect during cigarette smoking cessation. The observation that GST and MDA decreased in smoking rats given JOX-EO treatment compared to smoking rats suggests that the toxin can be reduced upon treatment with EOs. From the results, JOX-EO can reduce oxidative stress caused by cigarette smoke more than JPH-EO, administered at the same concentration. This study confirms the decrease in GSH, GPx, and CAT and an increase in GST and MDA associated with increased oxidative stress from cigarette smoke, despite the tests being done after 15 days of smoking cessation. The damages of oxidative stress may be long-lasting, which is why the system of a smoker takes a long time to get rid of the harmful effects of smoking, even after quitting smoking. According to the comparison between the effects of both EOs to reduce the toxicity of smoking, JOX-EO has an antioxidant capacity that can help the body return to its good health status faster after smoking cessation than JPH-EO. It was previously found that both EOs have antioxidant properties, with the JOX-EO having the highest antioxidant activity in in vitro studies [23]. This supports the favorable effect of JOX-EO in restoring RBC counts and improving the in vivo oxidative status in rat models. Medicinal plants are suitable therapies for diseases associated with cigarette smoking, as well as the depression and cognitive impairment that follow smoking cessation and nicotine withdrawal [57].

Studies on against cancer cell line A549 show that the EOs reduced cellular viability and induced cytotoxicity. Several studies have reported the anticancer effects of EOs against lung cancer, notably EOs from *Artemisia indica*, *Litsea cubeba*, *Tridax procumbens*, *Solanum spirale*, *Guatteria pogonopus*, *Malus domestica*, *Thymus vulgaris*, *Neolitsea variabillima*, *Comptonia peregrina*, and *Xylopia frutescens* [58–64]. Fruits from JOX and JPH, as well as their EOs, are commonly used in gastronomy and folk medicine to treat cancer and have demonstrated antioxidant and anticancer efficacy in experimental studies [65, 66]. The EOs under investigation are found to be rich in α-pinene, β-myrcene, and α-amorphene, as concerns JOX-EO, and tricyclene, α-pinene, and α-amorphene, as concerns JPH-EO [23]. Both EOs are abundantly rich in α-pinene, which has demonstrated antioxidant, anticancer, and genotoxic properties, and is also effective in treating respiratory tract infections [67, 68]. The ability of both EOs to boast in vivo oxidative parameters and inhibit lung cancer cell lines could be attributed to the abundant presence of α-pinene. This compound has been shown to possess antioxidant properties by inhibiting the generation of ROS, preventing lipid peroxidation, and inhibiting inflammatory mediators [69]. The compound α-pinene has also demonstrated the ability to inhibit in vivo metastatic lung colonization by cancer cells as well as apoptotic, antitumoral, antiproliferative, and cell cycle arrest capacities [70, 71]. Alpha-pinene is a monoterpene compound found in both EOs, which could have contributed significantly to the antioxidant and anticancer activity observed. This compound has the advantage that, despite being cytotoxic against cancer cells, it does not exhibit toxicity in normal cells and organs [72]. The mechanism of α-pinene's anticancer effects includes the induction of apoptosis, disruption of mitochondrial membrane potential, ROS production, increased Caspase 3 activity, heterochromatin aggregation, DNA fragmentation, exposure of phosphatidylserine, and reduction of tumor metastasis to the lungs in mice [70, 72, 73]. Biochemical observation in animal models revealed that α-pinene significantly mediated oxidant markers (nitrite and MDA) and antioxidant enzymes such as SOD, CAT, and GSH [74]. The results in this study suggest that α-pinene confers antioxidant, anti-inflammatory, and anticancer properties to the EOs. JOX-EO has demonstrated higher antioxidant and anticancer properties compared to JPH-EO. This could be attributed to the high amounts of β-myrcene previously detected in the JOX-EO, which is absent in the JPH-EO used in this study. β-myrcene has been shown to possess antioxidant and anti-inflammatory properties [75]. Additionally, β-myrcene is believed to possess anticancer effects, as evidenced by cytotoxicity, cell proliferation inhibition, cell migration inhibition, and morphological changes [76, 77]. EOs and their components, as well as other natural products, act synergistically to prevent various cancer cells and increase the efficacy of chemotherapy drugs [78, 79]. Therefore, the results of our study are of great importance, as the EOs of Juniper species can be used to reduce cigarette toxicants that are inhaled into the human body and also boast in vivo antioxidative parameters. Awareness of cigarette smoke toxicology and proper information about the toxic constituents found in smoke, as well as the illnesses they can cause, which include oxidative stress, cancer, respiratory diseases, heart disease, and stroke, can go a long way to change perceptions and cigarette cessation [80]. This study indicates the potential use of EOs in mitigating the harmful effects of cigarette smoke as a suitable alternative to other methods such as tobacco substitute sheets, dilution of smoke with glycerol, use of cigarette paper with high permeability, improved filter's filtration efficiency by split-tipping filters, or use of cellulose acetate filters.

## 5. Conclusion

This study presents a promising potential of JOX-EO and JPH-EO in mitigating the harmful effects of cigarette smoke. The EOs exhibited significant nicotine inhibition and reduced tar and PAHs. Moreover, the hematological parameters and oxidative stress markers indicate that the EOs, administered at 200 mg/kg for 15 days of postsmoking cessation, exhibit a profound capacity to speed up the recovery from the destructive effects of smoking. JOX-EO showed greater activity than JPH-EO. Both EOs exhibited promising cytotoxic potential against the A549 lung cancer cell line, surpassing that of etoposide, the standard used for comparison. These oils are rich in α-pinene, and their antioxidant and anticancer potentials can be attributed to this compound and others. Despite the challenges of oxidative stress persisting even after cessation, this research suggests that JOX-EO may contribute to a faster return to normal health in postsmoking situations, providing insights for potential therapeutic applications. Juniper EOs could be used to reduce cigarette smoke toxicants, improve antioxidant status, and reduce the risk of lung cancer associated with cigarette smoking.

## Figures and Tables

**Table 1:** Estimation of essential oil distillation time.

	Distilling time (min)	180	240	300	360
EOs yield %	JOX	0.54	0.56	0.57	0.58
	JPH	2.50	2.52	2.52	2.53

**Table 2:** Spectroscopic determination of nicotine extracted from tobacco and treated with essential oils.

Time of reaction	ABS of control	Conc. of nicotine in control solution μg/mL	Conc. (μg/mL)	Absorbance of samples in different concentration of EOs			
				JOX	JOX + N	JPH	JPH + N
5 min	0.319 ± 0.048	7.979 ± 1.268	19	0.170 ± 0.010	0.305 ± 0.011	0.156 ± 0.008	0.309 ± 0.005
			15.2	0.125 ± 0.006	0.289 ± 0.004	0.114 ± 0.009	0.307 ± 0.005
			11.4	0.090 ± 0.006	0.278 ± 0.008	0.081 ± 0.009	0.309 ± 0.007
			7.6	0.071 ± 0.003	0.306 ± 0.009	0.065 ± 0.012	0.316 ± 0.006
			3.8	0.063 ± 0.004	0.305 ± 0.008	0.050 ± 0.009	0.315 ± 0.011
			1.9	0.0006 ± 0.005	0.3101 ± 0.009	0.005 ± 0.004	0.318 ± 0.007

**Table 3:** Weight loss values of the neutral, alkaline, and acidic tar fractions treated with berry JOX and JPF essential oils (TBOX and TBPH) compared with controls.

Tar fractions	Tar rate (mg)		
	Control	TBOX	TBPH
Neutral	291.40 ± 8.80	135.67 ± 33.85	290.00 ± 4.00
Acidic	86.10 ± 4.20	60.00 ± 6.00	80.17 ± 5.39
Alkaline	64.80 ± 4.60	21.33 ± 4.16	60.00 ± 7.00

**Table 4:** Effect of JOX- and JPH-EOs on the relative organ (brain and lung) weight of Wistar rats. The results represent the mean ± SD of 4 determinations.

Groups		Final body weight (g)	Organ weight (g)		Relative organ weight %			
			Brain	Lung	Brain	*p* value	Lung	*p* value
			Mean ± SD	Mean ± SD	Mean ± SD		Mean ± SD	
Control groups	C	130.50 ± 15.19	1.56 ± 0.08	0.92 ± 0.09	1.23 ± 0.09	/	0.71 ± 0.02	/
	COX	148.00 ± 7.03	1.63 ± 0.06	0.93 ± 0.04	1.10 ± 0.02	0.24	0.63 ± 0.03	0.12
	CPH	154.00 ± 6.37	1.76 ± 0.04	0.97 ± 0.01	1.14 ± 0.02	0.43	0.63 ± 0.03	0.11
	CS	161.50 ± 5.78	1.64 ± 0.04	1.03 ± 0.01	1.01 ± 0.04	0.10	0.63 ± 0.02	0.08

Treated groups	STOX	152.00 ± 14.35	1.67 ± 0.03	1.06 ± 0.07	1.12 ± 0.07	0.40	0.69 ± 0.02	0.71
	STPH	152.75 ± 14.38	1.62 ± 0.03	0.99 ± 0.08	1.09 ± 0.12	0.39	0.65 ± 0.01	0.11

**Table 5:** Red blood cells (RBC), platelets (PLT), hemoglobin (HB), hematocrit (HCT), and mean corpuscular volume (MCV) values.

Red cell count	Control	STOX		STPH		CS		COX		CPH	
	Mean ± SD	Mean ± SD	*p* value	Mean ± SD	*p* value	Mean ± SD	*p* value	Mean ± SD	*p* value	Mean ± SD	*p* value
RBC (10^12^/L)	8.57 ± 0.25	8.55 ± 0.12	0.8648	11.35 ± 0.48	0.0005	12.27 ± 1.21	0.0010	6.50 ± 0.00	0.0003	7.25 ± 0.46	0.0024
PLT (10^9^/L)	1235.50 ± 8.81	1226.75 ± 4.03	0.1209	1293.50 ± 8.06	0.0006	1515.25 ± 104.36	0.0017	1210.50 ± 1.29	0.0013	1198.75 ± 6.85	0.0005
HB (g/dL)	15.45 ± 1.00	14.70 ± 0.31	0.2055	14.66 ± 0.13	0.1726	9.15 ± 1.65	0.0006	13.15 ± 0.50	0.0065	15.00 ± 0.54	0.4626
HCT %	44.00 ± 7.61	49.25 ± 0.95	0.2203	44.00 ± 4.39	> 0.999	29.25 ± 9.77	0.0547	45.25 ± 2.63	0.7668	44.75 ± 3.09	0.8612
MCV (fL)	55.75 ± 1.70	55.62 ± 1.10	0.9062	45.75 ± 2.50	0.0005	43.50 ± 2.38	0.0001	49.90 ± 2.16	0.0054	55.15 ± 0.87	0.5543

## Data Availability

The data that support the findings of this study are available on request from the corresponding author.
